# Fully automated volumetry of ventricular subregions on computed tomography using object detection and semantic segmentation

**DOI:** 10.1016/j.ynirp.2026.100325

**Published:** 2026-02-06

**Authors:** Raffaele Da Mutten, Olivier Zanier, Alessandro Carretta, Giorgio Palandri, Massimo Bottini, Daniel de Wilde, Ulf C. Schneider, Luca Regli, Carlo Serra, Victor E. Staartjes

**Affiliations:** a Machine Intelligence in Clinical Neuroscience and Microsurgical Neuroanatomy (MICN) Laboratory, Department of Neurosurgery, Clinical Neuroscience Center, University Hospital Zurich, University of Zurich, Zurich, Switzerland; b Department of Neurosurgery, Cantonal Hospital Lucerne, Lucerne, Switzerland; c Department of Oncology, University of Oxford, Oxford, United Kingdom; d Department of Biomedical and NeuroMotor Sciences (DIBINEM), University of Bologna, Italy; e Programma Neurochirurgia dell’Ipofisi - Pituitary Unit, IRCCS delle Scienze Neurologiche di Bologna, Bologna, Italy; f Unit of Neurosurgery, IRCCS Instituto delle Scienze Neurologiche di Bologna, 40139, Bologna, Italy; g Department of Clinical Neuroscience, Karolinska Institutet, Stockholm, Sweden; h Capio Spine Center Stockholm, Löwenströmska Hospital, Upplands-Väsby, Sweden

**Keywords:** Machine learning, Deep learning, Neurosurgery, Artificial intelligence, Algorithms, Hydrocephalus

## Abstract

**Background:**

Our goal was to develop and validate machine learning models that are capable of fully automatic identification and segmentation of frontal, temporal, and posterior horns, the body of the lateral ventricle, the third and fourth ventricle, as well as the atrium on either side.

**Methods:**

Patients shunted for hydrocephalus were included. Data from two centers was used for development/external validation, respectively. Manual labelling of ventricular subregions on computed tomography (CT) was performed. First, an object detection algorithm (YOLOv5) was trained. This allowed for precise cropping of the subregions that could then be used as input for a 2D U-Net. For comparison, a nnU-Net was also trained. Precision, recall, mean average precision 50 and 50–95 (mAP50; mAP50-95) were used as performance metrics for the YOLO algorithm. Dice score, Jaccard score, and 95th percentile Hausdorff distance assessed performance for the U-Net.

**Results:**

80 CTs from patients at our center were included, as well as 50 from a second center. The mean age was 68.59 ± 15.89 and 75.94 ± 4.17 for the first and second centers, and 43 (52.5%) and 30 (60%) were male. MAP 50, mAP50-95 was 0.728, 0.453 for internal and 0.274, 0.124 for external validation across all classes. Best mean Dice scores were 0.92 ± 0.1 and 0.90 ± 0.05 for the body of the left lateral ventricle.

**Conclusions:**

Automatic segmentation and volumetry of ventricles including their subregions was feasible with high precision on computed tomography, potentially helping the clinical evaluation of even subtle changes in ventricular volume.

## Highlights

• Our pipeline enables reliable automated volumetry of anatomical subregions of the internal ventricular system.
• Combining object detection with semantic segmentation improves accuracy of ventricular subregion segmentation.
• A user-friendly graphical interface facilitates clinical integration of the deep learning pipeline.

## Abbreviations:

CT: computed tomography
CSF: cerebrospinal fluid
NPH: normal pressure hydrocephalus
ICC: intraclass correlation coefficient
MRI: magnetic resonance imaging
RVE: relative volumetric error

## 1 Introduction

Hydrocephalus is a condition described as early as Hippocrates and Galen (Aschoff et al., 1999). It is generally defined as an imbalance of cerebrospinal fluid (CSF) production and absorption (Rekate, 2009). Next to MRI, CT is a valuable diagnostic tool. Etiologies include congenital, post-traumatic, hemorrhage, infections, and idiopathic (Hochstetler et al., 2022).

CSF volume is also a key diagnostic feature in normal pressure hydrocephalus (NPH), with patients commonly obtaining serial scans. Numerous indices indicative of NPH have been created and validated. The well-known Evan's index shows an intraclass correlation coefficient (ICC) of 0.81–0.913, while the temporal horn width is described to have an ICC of 0.729 (Evans, 1942; Miskin et al., 2017; Reinard et al., 2015).

Subtle changes over time can be difficult to detect manually, also due to inconsistent positioning of the head in CTs. Ryska et al. (2020) showed that the angulation of the section plane can strongly influence the diagnostic performance of many radiological indices for NPH. This study was performed on magnetic resonance imaging (MRI) scans, yet since CT generally has a less standardized acquisition frame compared to MRI, these results might also apply to CT. In a smaller cohort, Toma et al. (2011) investigated this question for CT, concluding that plane selection largely influences results. Also, Lo et al. (2021), as well as Han et al. (2022), identified unique topological and volumetric features of NPH. Hence, volumetry seems beneficial compared to manual linear measurements (Ambarki et al., 2010; Aukland et al., 2008; Toma et al., 2011).

We hypothesize that deploying fully automated image segmentation can provide valuable information about disease identification and progression. Utilizing three-dimensional information can omit the question of section plane angulation. Also, the quantification of anatomical subregions allows for the exploration of new indices based on the shape and geometry of the regions.

Advances in machine learning in terms of inference speed and precision have increased the utility of such approaches (King, 2018; Obermeyer and Emanuel, 2016). Consequently, we hope to create a useful tool for clinicians, offering better correlation to true ventricular volume, increased objectivity, and lastly, more quick and valuable clinical information by providing three-dimensional measurements instead of the usual ventricular width only.

## 2 Methods

### 2.1 Data collection

From our center (University Hospital of Zurich, Switzerland), we collected CT scans of 80 patients with hydrocephalus due to different pathologies, including mainly normal pressure hydrocephalus and posthemorrhagic hydrocephalus. To ensure generalizability, we validated the models on a second external validation set of 50 CT scans from another center (University Hospital of Bologna, Italy). Those were only patients with normal pressure hydrocephalus.

### 2.2 Ethical considerations

Patient data were handled in accordance with the ethical standards outlined in the Declaration of Helsinki and its amendments. The use of this data received approval from the institutional review boards in Zurich (IRB, Cantonal Ethics Committee Zürich, BASEC, 2023-00688). For data collected in Bologna, ethical approval was granted by the ethics committee of the greater area of Emilia-Romagna, Italy (No 94-2025-OSS-AUSLBO).

### 2.3 Labelling

Images were labelled by hand by a neurosurgical resident (RDM) and two medical students (MB, DDW; masks were checked by another rater and discussed with an independent reviewer when in disagreement). The anterior horn, body of the lateral ventricle, atrium, posterior horn, temporal horn, third and fourth ventricle were delineated individually using 3D Slicer (Fedorov et al., 2012). Standard anatomical landmarks were considered as boundaries for the subregions when they were visible on axial CT (Mortazavi et al., 2014).

### 2.4 Image preprocessing

First, images were converted into the NIfTI (Neuroimaging Informatic Technology Initiative) format (Li et al., 2016). Second, voxels were resampled to an extension of isotropic 1.0 × 1.0 × 1.0 mm and a total size of 256 × 256 x 256. Hounsfield units were windowed from 0 to 80 for optimal contrast of cerebrospinal fluid and ependyma (CSF window). For computational efficiency, images were sliced in axial orientation. As the two-stage approach created specific regions of interest and thereby provides highly homogenized input images for the semantic segmentation models, no further preprocessing steps were undertaken.

### 2.5 Object detection

A YOLOv5 (You Look Only Once) algorithm was trained to detect the before-mentioned individual subregions (Jocher et al., 2020). Training was carried out for 300 epochs, with a batch size of 64 and an image size of 256 × 256, using data augmentation that included flipping, scaling, and translation. Training was carried out on an NVIDIA RTX A6000 with 48 GB of memory.

Subsequently, the CT slices were cropped using the bounding boxes used for training before. Since they did not fit the input layer of the subsequent U-Net, they were enlarged by up to 300% and padded, resulting in standardized 256 × 256 slices. This allowed for the more specific training of segmentation networks that only include the respective anatomical subregion.

### 2.6 Semantic segmentation

From the cropped CT slices and their accordingly processed mask, eleven models were trained. A standard U-Net architecture was chosen (Ronneberger et al., 2015). For each of the subregions – left anterior horn, right anterior horn, third ventricle, etc. – a specific model was trained in five-fold cross-validation. Hyperparameter tuning included the number of starting neurons, ranging from 32 to 128, the net depth (from 3 to 5), the number of blocks (from 2 to 5), dropout functions, batch normalization, batch size (32–64), and the learning rate (0.001 or 0.0001). A rectified linear unit was constantly chosen as activation function and binary crossentropy as loss function. Since the posterior horn only had a small number of input slices, right and left were combined for data augmentation. Additionally, all models were trained using an enlarged dataset by flipped, shifted, rotated, and brightness-adjusted copies of the training data. Training was carried out on an NVIDIA RTX 3090 graphical processing unit with 32 GB of memory.

### 2.7 Evaluation

The object detection algorithm used to create subregions was evaluated using recall, precision, mean average precision (mAP) 50 as well as mAP50-95 (Reis et al., 2023).

Dice similarity coefficient, Jaccard coefficient and 95th percentile Hausdorff distance were used to assess the performance of the semantic segmentation models (Dice, 1945; Jaccard, 1912; Ralescu, 1984; Taha and Hanbury, 2015). Those metrics compared manually segmented labels with predicted masks. The first two are a measure for overlap ranging from zero to one, the latter analyzes the distance between the edges of the segmentation. Hence, high values for Dice and Jaccard scores mean good congruence and small for Hausdorff distance respectively. While training validation used a different subset of slices for each round of cross-validation, all models from the holdout set were assessed on the same set of slices that was put aside before training. On the 50 CT scans from the external dataset, the entire pipeline was tested as external validation. Additionally, volumes between the ground truth data and the predicted masks were compared for all subregions, the entire internal ventricular system, the left and right side (frontal horn, body of lateral horn, atrium, and temporal horn) as well as the midline regions (third and fourth ventricle). Subsequently, the relative volumetric error from these volumes was extrapolated.

### 2.8 Backsampling

**Fig. 1:**
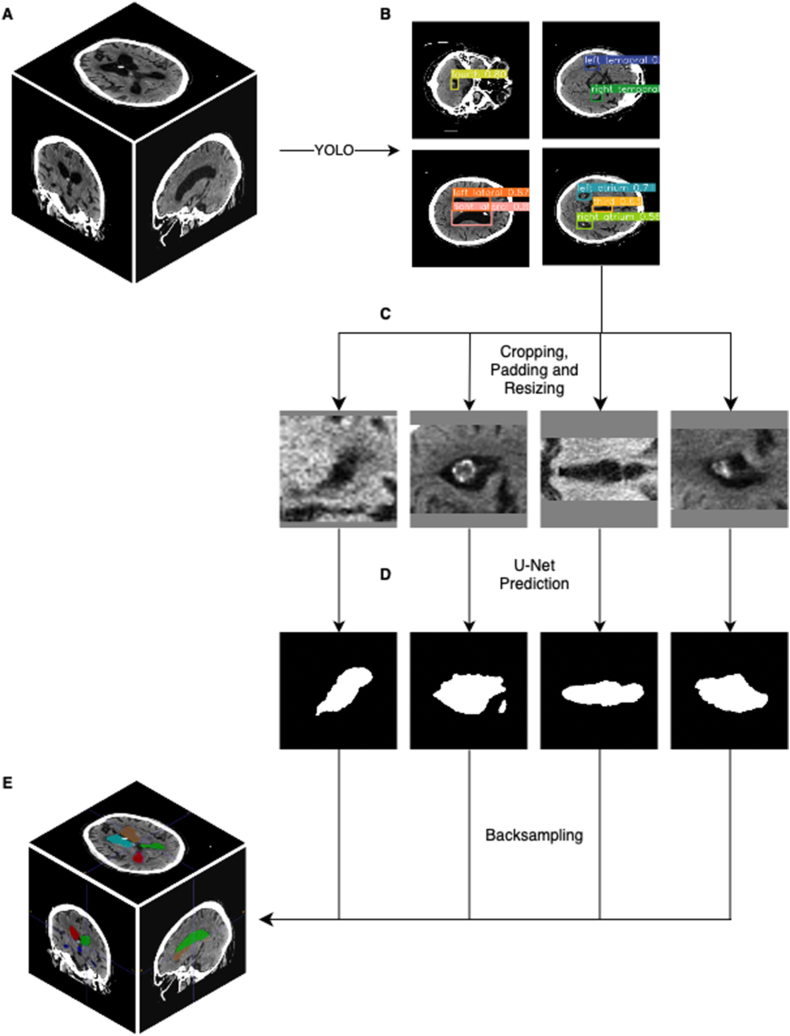
Schematic illustration of the inference pipeline: (A) The initial CT scan is normalized, resampled and sliced in axial direction. (B) The YOLO model detects the anatomical subregions. (C) Axial slices are then cropped, padded, and resized according to the created bounding boxes. (D) The respective U-Net for each anatomical subregion creates a prediction. (E) Lastly, these predictions are uncropped, unpadded and resized into their original dimension and combined into one final segmentation.

In order to achieve coherent three-dimensional volumes from the disassembled U-Net outputs, the slides were reformatted into their original dimensions using the bounding box as guide. This included uncropping, resizing and then stacking the two-dimensional images into the correct position in three-dimensional space. The entire process is illustrated in Fig. 1.

### 2.9 Deployment

**Supplementary Fig. 1:**
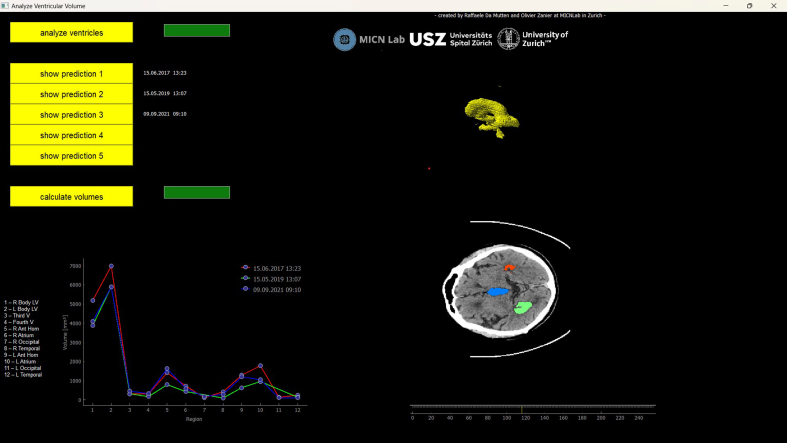
Exemplary demonstration of the application of the pipeline in the graphical user interface.

For easy access, a graphical user interface was created where the raw DICOM files can be used directly. The graphical user interface transforms the single DICOM slices to one NIfTI per series, creates an intensity window around the CSF and resizes the image to 256 × 256 × 256, identical to the training data (Li et al., 2016). Then, the pipeline is applied. The resulting segmentation mask is then overlayed over the input image and displayed. The resulting appearance can be seen in Supplementary Figure 1.

### 2.10 nnU-Net

To compare performance, we also trained a widely applied architecture, the self-configuring NN-UNet on our data without using the two-stage approach. In the same manner, we performed internal and external validation using the same metrics.

## 3 Results

### 3.1 Dataset

80 patients from the first center were included. Demographics and etiologies of hydrocephalus can be found in Table 1.

**Table 1:** Summary of the patient demographics and characteristics.

Center	Zurich	Bologna
**Baseline**		

Number of Patients	80	50 (100)
Age years, Mean ± SD	68.59 ± 15.89	75.94 ± 4.17
Male gender, n (%)	43 (52.5)	30 (60)

**Pathology**		

Normal Pressure Hydrocephalus, n (%)	27 (33.8)	50 (100)
Subarachnoid Bleeding, n (%)	26 (32.5)	0 (0)
Trauma, n (%)	7 (8.8)	0 (0)
Intracranial Bleeding, n (%)	4 (5.0)	0 (0)
Unclear, n (%)	5 (6.2)	0 (0)
Cerebellar Bleeding, n (%)	1 (1.2)	0 (0)
Chiari Malformation Type 2, n (%)	1 (1.2)	0 (0)
Plexus Papilloma, n (%)	1 (1.2)	0 (0)
Meningoencephalitis, n (%)	1 (1.2)	0 (0)
Trauma with Subdural Hematoma, n (%)	1 (1.2)	0 (0)

**Revision Surgery**		

No, n (%)	65 (81.2)	50 (100)
Yes, n (%)	15 (18.8)	0 (0)

**Shunt Type**		

Ventriculoperitoneal, n (%)	79 (98.8)	50 (100)
Ventriculoatrial, n (%)	1 (1.2)	0 (0)

**Volume**				
	Mean ± SD	Median (IQR)	Mean ± SD	Median (IQR)
Body of Right Lateral Ventricle, ml	32.4 ± 26.63	25.5 (22.5)	37.59 ± 15.72	35.22 (17.45)
Body of Left Lateral Ventricle, ml	33.5 ± 24.38	26.0 (27.0)	41.16 ± 17.12	37.53 (19.73)
Third Ventricle, ml	3.16 ± 2.02	3.0 (2.0)	3.25 ± 1.02	3.18 (1.08)
Fourth Ventricle, ml	2.02 ± 1.71	2.0 (2.0)	2.10 ± 0.80	2.14 (0.99)
Right Anterior Horn, ml	8.62 ± 4.41	8.0 (5.0)	4.94 ± 2.79	4.31 (4.07)
Right Atrium, ml	5.7 ± 4.51	5.0 (4.0)	6.19 ± 2.92	5.88 (3.38)
Right Posterior Horn, ml	0.7 ± 1.46	0.0 (1.0)	1.31 ± 1.22	0.97 (1.47)
Right Temporal Horn, ml	3.51 ± 2.60	3.0 (3.0)	2.21 ± 1.17	1.90 (0.97)
Left Anterior Horn, ml	9.44 ± 4.55	8.5 (6.3)	5.20 ± 3.07	4.54 (3.81)
Left Atrium, ml	6.64 ± 4.82	6.0 (4.0)	7.65 ± 3.82	6.87 (6.15)
Left Posterior Horn, ml	1.26 ± 2.57	0.0 (1.0)	1.97 ± 1.76	1.49 (2.46)
Left Temporal Horn, ml	3.76 ± 2.57	3.0 (3.0)	2.41 ± 1.23	2.05 (1.58)

SD, standard deviation; IQR, interquartile range.

For external validation, 50 cases of patients with normal pressure hydrocephalus who underwent surgery for a ventriculoperitoneal shunt were included.

### 3.2 YOLO: object detection

The training dataset included 9565 slices, the training validation dataset 2291 slices (Table 2: Train) and the hold-out dataset after training (Table 2: Hold) 2120 slices. The external validation dataset was made up of 12800 slices. The occurrence of each subregion on these slices is reported in Table 2 under instances.

**Table 2:** Object detection algorithm (YOLO) performance on all three datasets (Train: training validation; Hold: holdout set; Ext: external validation), comparing predicted boxes and boxes created from manual segmentations.

Measurement	Instances			Precision			Recall			mAP50			mAP50 - 95		
Dataset	Train	Hold	Ext	Train	Hold	Ext	Train	Hold	Ext	Train	Hold	Ext	Train	Hold	Ext
Structure															
All	2385	2762	11203	0.700	0.767	0.420	0.727	0.732	0.301	0.682	0.728	0.274	0.431	0.453	0.124
Body of Right Lateral Ventricle	458	495	1646	0.810	0.816	0.290	0.558	0.558	0.549	0.603	0.581	0.332	0.449	0.437	0.155
Body of Left Lateral Ventricle	326	322	1652	0.825	0.812	0.266	0.866	0.899	0.343	0.885	0.917	0.165	0.685	0.659	0.076
Third Ventricle	171	195	681	0.784	0.703	0.213	0.713	0.763	0.011	0.727	0.701	0.029	0.424	0.382	0.010
Fourth Ventricle	209	236	801	0.818	0.863	0.607	0.699	0.797	8.700	0.788	0.825	0.680	0.456	0.535	0.350
Right Anterior Horn	215	251	697	0.751	0.768	0.298	0.814	0.773	0.007	0.725	0.746	0.055	0.511	0.423	0.027
Right Atrium	136	186	959	0.564	0.677	0.535	0.684	0.785	0.345	0.568	0.712	0.368	0.372	0.444	0.162
Right Posterior Horn	39	48	652	0.469	0.859	0.192	0.538	0.542	0.353	0.523	0.605	0.242	0.173	0.289	0.083
Right Temporal Horn	205	276	849	0.778	0.764	0.327	0.787	0.797	0.432	0.770	0.797	0.372	0.46	0.519	0.168
Left Anterior Horn	197	240	681	0.783	0.823	0.673	0.933	0.812	0.119	0.804	0.779	0.154	0.615	0.529	0.092
Left Atrium	160	208	980	0.698	0.768	0.802	0.779	0.745	0.050	0.738	0.818	0.272	0.493	0.545	0.110
Left Posterior Horn	44	56	750	0.384	0.642	0.377	0.591	0.446	0.307	0.301	0.441	0.265	0.071	0.178	0.103
Left Temporal Horn	225	249	855	0.733	0.715	0.456	0.760	0.871	0.399	0.757	0.817	0.350	0.458	0.496	0.150

mAP50, Mean Average Precision with Jaccard Threshold of 0.5; MAP with 10 Threshold steps between 0.5 and 0.95.

The final model consists of 416 layers and 140 million parameters. For validation, an intersection over union threshold of 0.6 and a confidence threshold of 0.001 was used.

The final YOLOv5 model reached a precision of 0.7, recall of 0.727, mAP50 of 0.682 and mAP50-95 of 0.431 for all classes in training. Internal validation achieved respective values of 0.767, 0.732, 0.728 and 0.453. External validation was evaluated at 0.42, 0.301, 0.274 and 0.124. Detailed results for all individual classes can be seen in Table 2.

### 3.3 U-Nets: semantic segmentation

Detailed results for U-Net performance are reported in Table 3. The best Dice score for training validation was the body of the left lateral ventricle with a Dice score (mean ± standard deviation) of 0.92 ± 0.08, 0.91 ± 0.1 for internal validation and 0.90 ± 0.05 for external validation. Respective Jaccard scores were 0.87 ± 0.1, 0.85 ± 0.12 and 0.82 ± 0.07. The nnU-Net yielded a mean Dice score of 0.74 ± 0.34, 0.74 ± 0.34 and 0.41 ± 0.23 for the according cohorts, as in Table 4.

**Table 3:** **YOLO and 2D-U-Net:** Training validation, holdout and external validation performance of manual segmentations versus predictions.

	Training Validation		Holdout Validation		External Validation	
	Mean ± SD	Median (IQR)	Mean ± SD	Median (IQR)	Mean ± SD	Median (IQR)
**Dice**						

Body of Right Lateral Ventricle	0.87 ± 0.18	0.93 (0.071)	0.89 ± 0.14	0.93 (0.063)	0.89 ± 0.05	0.90 (0.04)
Body of Left Lateral Ventricle	0.92 ± 0.08	0.95 (0.04)	0.91 ± 0.1	0.94 (0.045)	0.90 ± 0.05	0.91 (0.04)
Third Ventricle	0.8 ± 0.17	0.85 (0.111)	0.82 ± 0.12	0.84 (0.119)	0.73 ± 0.12	0.75 (0.10)
Fourth Ventricle	0.8 ± 0.1	0.82 (0.118)	0.78 ± 0.24	0.87 (0.144)	0.75 ± 0.07	0.77 (0.08)
Right Anterior Horn	0.88 ± 0.1	0.9 (0.065)	0.89 ± 0.07	0.9 (0.052)	0.77 ± 0.17	0.83 (0.16)
Right Atrium	0.75 ± 0.2	0.83 (0.175)	0.85 ± 0.07	0.87 (0.095)	0.66 ± 0.16	0.69 (0.18)
Right Temporal Horn	0.8 ± 0.2	0.86 (0.106)	0.8 ± 0.18	0.86 (0.11)	0.61 ± 0.18	0.66 (0.22)
Left Anterior Horn	0.91 ± 0.05	0.93 (0.044)	0.91 ± 0.05	0.92 (0.043)	0.78 ± 0.17	0.81 (0.17)
Left Atrium	0.9 ± 0.06	0.91 (0.047)	0.89 ± 0.04	0.9 (0.054)	0.64 ± 0.13	0.67 (0.15)
Posterior Horns	0.18 ± 0.24	0.08 (0.303)	0.43 ± 0.17	0.43 (0.269)	0.18 ± 0.05	0.18 (0.03)
Left Temporal Horn	0.83 ± 0.18	0.88 (0.091)	0.81 ± 0.14	0.86 (0.126)	0.62 ± 0.17	0.69 (0.20)

**Jaccard**						

Body of Right Lateral Ventricle	0.8 ± 0.2	0.87 (0.121)	0.81 ± 0.16	0.87 (0.109)	0.81 ± 0.08	0.82 (0.07)
Body of Left Lateral Ventricle	0.87 ± 0.1	0.9 (0.072)	0.85 ± 0.12	0.89 (0.08)	0.82 ± 0.07	0.84 (0.07)
Third Ventricle	0.7 ± 0.18	0.74 (0.165)	0.71 ± 0.14	0.73 (0.178)	0.58 ± 0.12	0.60 (0.12)
Fourth Ventricle	0.68 ± 0.13	0.7 (0.168)	0.68 ± 0.24	0.77 (0.216)	0.61 ± 0.09	0.63 (0.10)
Right Anterior Horn	0.79 ± 0.13	0.82 (0.107)	0.81 ± 0.09	0.82 (0.086)	0.66 ± 0.18	0.71 (0.22)
Right Atrium	0.64 ± 0.21	0.7 (0.243)	0.75 ± 0.1	0.76 (0.145)	0.50 ± 0.15	0.52 (0.20)
Right Temporal Horn	0.7 ± 0.19	0.75 (0.161)	0.7 ± 0.19	0.75 (0.165)	0.46 ± 0.18	0.49 (0.23)
Left Anterior Horn	0.84 ± 0.07	0.86 (0.075)	0.84 ± 0.08	0.86 (0.073)	0.66 ± 0.19	0.69 (0.24)
Left Atrium	0.82 ± 0.08	0.84 (0.079)	0.81 ± 0.07	0.83 (0.088)	0.48 ± 0.13	0.50 (0.17)
Posterior Horns	0.12 ± 0.18	0.04 (0.179)	0.29 ± 0.14	0.27 (0.221)	0.10 ± 0.03	0.10 (0.02)
Left Temporal Horn	0.73 ± 0.17	0.78 (0.14)	0.71 ± 0.16	0.75 (0.187)	0.47 ± 0.17	0.52 (0.22)

**95**th **percentile Hausdorff distance**						

Body of Right Lateral Ventricle	14.82 ± 21.8	6.84 (7.649)	12.84 ± 15.74	6.4 (9.701)	4.2 ± 2.45	3.46 (3.0)
Body of Left Lateral Ventricle	7.88 ± 8.66	5.1 (4.062)	9.21 ± 10.05	5.39 (5.96)	5.08 ± 4.12	4.0 (3.33)
Third Ventricle	15.71 ± 13.63	9.86 (17.041)	12.81 ± 10.3	9.86 (10.809)	5.98 ± 7.43	4.36 (2.66)
Fourth Ventricle	15.39 ± 12.87	9.0 (17.01)	14.96 ± 17.51	6.4 (12.08)	3.61 ± 3.78	2.95 (1.33)
Right Anterior Horn	13.47 ± 14.32	10.16 (7.746)	9.86 ± 5.58	8.56 (5.98)	3.79 ± 5.87	2.24 (1.58)
Right Atrium	24.65 ± 25.85	16.01 (19.097)	20.14 ± 10.72	19.39 (10.534)	6.69 ± 4.03	5.74 (3.23)
Right Temporal Horn	11.55 ± 16.7	6.4 (5.63)	11.58 ± 15.38	6.12 (6.949)	12.98 ± 11.29	10.18 (15.81)
Left Anterior Horn	8.59 ± 5.74	7.07 (4.193)	7.43 ± 3.5	6.38 (3.355)	3.46 ± 5.25	2.0 (2.2)
Left Atrium	11.25 ± 8.02	8.05 (6.504)	13.0 ± 7.28	11.0 (11.333)	6.56 ± 3.05	6.0 (4.0)
Posterior Horns	143.19 ± 71.6	145.46 (150.05)	138.32 ± 13.96	140.37 (17.329)	17.06 ± 0.66	17.06 (0.66)
Left Temporal Horn	12.22 ± 12.65	7.21 (8.463)	11.84 ± 14.9	7.07 (7.37)	13.53 ± 10.27	10.44 (16.92)

SD, standard deviation; IQR, interquartile range.

**Table 4:** nn**U-Net:** Validation Training, holdout and external validation performance of manual segmentations versus predictions.

	Validation Training		Holdout Validation		External Validation	
	Mean ± SD	Median (IQR)	Mean ± SD	Median (IQR)	Mean ± SD	Median (IQR)
**Dice**						

Body of Right Lateral Ventricle	0.73 ± 0.33	0.88 (0.12)	0.73 ± 0.33	0.88 (0.12)	0.50 ± 0.21	0.51 (0.32)
Body of Left Lateral Ventricle	0.74 ± 0.34	0.90 (0.09)	0.74 ± 0.34	0.90 (0.09)	0.41 ± 0.23	0.42 (0.43)
Third Ventricle	0.82 ± 0.11	0.87 (0.09)	0.82 ± 0.11	0.87 (0.09)	0.21 ± 0.28	0.00 (0.50)
Fourth Ventricle	0.85 ± 0.05	0.87 (0.06)	0.85 ± 0.05	0.87 (0.06)	0.20 ± 0.26	0.00 (0.47)
Right Anterior Horn	0.72 ± 0.28	0.81 (0.19)	0.72 ± 0.28	0.81 (0.19)	0.41 ± 0.27	0.45 (0.43)
Right Atrium	0.58 ± 0.32	0.72 (0.37)	0.58 ± 0.32	0.72 (0.37)	0.02 ± 0.07	0.00 (0.00)
Right Posterior Horn	0.27 ± 0.36	0.00 (0.67)	0.27 ± 0.36	0.00 (0.67)	0.01 ± 0.05	0.00 (0.00)
Right Temporal Horn	0.61 ± 0.33	0.76 (0.42)	0.61 ± 0.33	0.76 (0.42)	0.06 ± 0.13	0.00 (0.04)
Left Anterior Horn	0.70 ± 0.29	0.81 (0.27)	0.70 ± 0.29	0.81 (0.27)	0.43 ± 0.26	0.44 (0.38)
Left Atrium	0.54 ± 0.32	0.68 (0.47)	0.54 ± 0.32	0.68 (0.47)	0.02 ± 0.07	0.00 (0.00)
Left Posterior Horn	0.27 ± 0.35	0.00 (0.61)	0.27 ± 0.35	0.00 (0.61)	0.02 ± 0.08	0.00 (0.00)
Left Temporal Horn	0.58 ± 0.36	0.77 (0.60)	0.58 ± 0.36	0.77 (0.60)	0.20 ± 0.21	0.09 (0.38)

Jaccard						

Body of Right Lateral Ventricle	0.65 ± 0.31	0.78 (0.19)	0.65 ± 0.31	0.78 (0.19)	0.36 ± 0.18	0.34 (0.29)
Body of Left Lateral Ventricle	0.67 ± 0.32	0.82 (0.15)	0.67 ± 0.32	0.82 (0.15)	0.28 ± 0.19	0.26 (0.35)
Third Ventricle	0.71 ± 0.14	0.77 (0.13)	0.71 ± 0.14	0.77 (0.13)	0.15 ± 0.20	0.00 (0.33)
Fourth Ventricle	0.75 ± 0.07	0.77 (0.09)	0.75 ± 0.07	0.77 (0.09)	0.14 ± 0.19	0.00 (0.30)
Right Anterior Horn	0.61 ± 0.26	0.69 (0.27)	0.61 ± 0.26	0.69 (0.27)	0.29 ± 0.22	0.29 (0.35)
Right Atrium	0.47 ± 0.28	0.56 (0.39)	0.47 ± 0.28	0.56 (0.39)	0.01 ± 0.04	0.00 (0.00)
Right Posterior Horn	0.21 ± 0.30	0.00 (0.50)	0.21 ± 0.30	0.00 (0.50)	0.01 ± 0.03	0.00 (0.00)
Right Temporal Horn	0.51 ± 0.31	0.62 (0.48)	0.51 ± 0.31	0.62 (0.48)	0.03 ± 0.08	0.00 (0.02)
Left Anterior Horn	0.60 ± 0.28	0.68 (0.37)	0.60 ± 0.28	0.68 (0.37)	0.30 ± 0.21	0.29 (0.33)
Left Atrium	0.43 ± 0.29	0.52 (0.45)	0.43 ± 0.29	0.52 (0.45)	0.01 ± 0.04	0.00 (0.00)
Left Posterior Horn	0.22 ± 0.29	0.00 (0.44)	0.22 ± 0.29	0.00 (0.44)	0.01 ± 0.05	0.00 (0.00)
Left Temporal Horn	0.48 ± 0.31	0.62 (0.57)	0.48 ± 0.31	0.62 (0.57)	0.13 ± 0.14	0.05 (0.23)

95th percentile Hausdorff distance						

Body of Right Lateral Ventricle	11.26 ± 13.14	5.05 (7.79)	11.26 ± 13.14	5.05 (7.79)	32.69 ± 25.13	24.33 (22.36)
Body of Left Lateral Ventricle	10.81 ± 12.78	4.74 (6.00)	10.81 ± 12.78	4.74 (6.00)	45.98 ± 33.56	32.40 (65.13)
Third Ventricle	3.81 ± 3.28	2.40 (2.23)	3.81 ± 3.28	2.40 (2.23)	5.69 ± 14.29	0.00 (5.74)
Fourth Ventricle	2.01 ± 0.84	1.98 (1.13)	2.01 ± 0.84	1.98 (1.13)	7.85 ± 14.26	3.61 (8.27)
Right Anterior Horn	7.43 ± 7.87	3.80 (4.75)	7.43 ± 7.87	3.80 (4.75)	18.20 ± 25.13	8.60 (12.79)
Right Atrium	17.26 ± 19.82	5.58 (29.39)	17.26 ± 19.82	5.58 (29.39)	6.22 ± 13.11	0.00 (8.00)
Right Posterior Horn	8.43 ± 14.50	0.50 (9.71)	8.43 ± 14.50	0.50 (9.71)	18.11 ± 29.05	0.00 (39.16)
Right Temporal Horn	17.39 ± 24.24	4.71 (15.91)	17.39 ± 24.24	4.71 (15.91)	25.49 ± 32.85	14.32 (29.51)
Left Anterior Horn	7.29 ± 7.50	3.56 (7.77)	7.29 ± 7.50	3.56 (7.77)	9.92 ± 8.33	8.00 (7.62)
Left Atrium	15.72 ± 18.73	5.70 (11.81)	15.72 ± 18.73	5.70 (11.81)	15.32 ± 24.06	0.00 (22.10)
Left Posterior Horn	11.59 ± 18.47	2.70 (8.90)	11.59 ± 18.47	2.70 (8.90)	9.92 ± 30.41	0.00 (0.00)
Left Temporal Horn	19.59 ± 24.51	5.27 (28.81)	19.59 ± 24.51	5.27 (28.81)	28.25 ± 25.46	22.62 (50.78)

SD, standard deviation; IQR, interquartile range.

Lowest scores were obtained for the posterior horns with a mean Dice score of 0.18 ± 0.24, 0.43 ± 0.17 and 0.18 ± 0.05 for training validation, internal and external validation.

**Supplementary Fig. 2:**
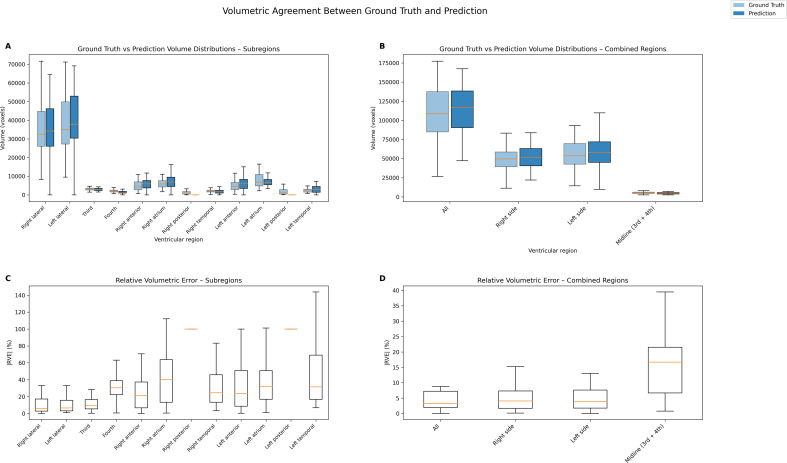
Volumetric agreement between ground truth and predicted ventricular segmentations. (**A–B)** Distribution of ventricular subregion and combined-region volumes for ground truth and predictions. **(C**–**D)** Relative volumetric error (RVE) for the same regions.

Supplementary Fig. 2 illustrates the volumetric performance for the external validation dataset.

## 4 Discussion

We developed a ready-to-use pipeline that estimates the ventricular volume of anatomical subregions on the basis of a brain CT scan. Using machine learning, an otherwise laborious task of segmenting on each slice by hand can be simplified time-efficiently.

Both the bounding box and the semantic segmentation model showed good performance at internal and external validation for most subregions. The YOLO model showed mAP50 of 0.682, 0.728 and 0.274 for training validation, internal holdout set and external validation. The lower performance on new and unseen data is expected and can be compensated by choosing a low detection threshold. This ensures that no slices are missed.

The semantic segmentation models performed satisfactorily in most cases as well. Especially large and frequently occurring subregions, such as the body of the lateral ventricle, had excellent results. Naturally, regions with lower occurrences in the training data faired inferior in terms of metric performance compared to more common ones. This was especially notable for the posterior horns, reflected by only 39–56 instances in the training validation and holdout set of the YOLO model. This limits the number of input slices for the following U-Net. External validation results also seem generally lower. While this is an expected tendency in machine learning models, some loss of performance is probably attributed to the fact that the entire pipeline was used for external validation, thereby introducing some additional error. Notably, the transitions between subregions can diminish performance as labelling and predictions were only executed on axial slices. This is not always perfectly aligned with anatomical boundaries in the ventricular system. This is also illustrated by the higher relative volumetric error (RVE) of individual subregions compared to combinations such as the entire left side. For future projects, combining all three planes could potentially help alleviate that issue. Smaller regions, such as the third and fourth ventricles, and especially the posterior horn, showed higher RVE. As the RVE for the entire ventricular system was relatively low, the possibility to answer the clinically relevant questions of width and changes over time should not be affected by that.

Toma et al. stated that the strength of correlation between radiological indexes and the actual ventricular volume depends heavily on the slice that is chosen for assessment (Toma et al., 2011). Hence, integrating the measurements of multiple slices and thereby calculating the volume should be beneficiary.

Our fully automated approach offers several clinical benefits. First, ventricular width is a highly important feature in many pathologies. While it can be objectified by attempting to reproduce the same slice orientation in three-dimensionally reconstructed CT scans, inaccuracies can easily occur. Automation of assessment ensures reproducibility and objectivity. Second, volumetry is more sensitive to changes than linear measurements, as shown for tumors (Cai and Hong, 2018; Ellingson et al., 2021). Third, certain types of hydrocephalus, especially the occlusive type, can only affect partial subregions of the ventricular system. This is novel in our pipeline: subregions and also temporal changes can be quantified.

Furthermore, the CT scans were not aligned, and more than one CT vendor was included. The resulting more heterogeneous dataset should make the models more robust and increase their generalizability. As our primary goal was to design a pipeline fit for clinical use, the dataset included two common vendors (GE and Philips). Nonetheless, external validation performance using the entire pipeline was very adequate for most cases and not notably lower than internal validation.

Other similar work, that included fewer subregions, reached Dice scores of up to 0.95 (Huff et al., 2019; Klimont et al., 2019; Maragkos et al., 2021). Huff et al. did not perform external validation and only included non-pathological cases (Huff et al., 2019). While Maragkos et al. used a more diverse dataset, also no external validation was performed on a separately obtained dataset (Maragkos et al., 2021). Goo HW used a semi-automatic threshold-based approach for ventricular volume quantification, requiring 10–15 min per scan (Goo, 2021). Our pipeline can accelerate this value to roughly 3 min per scan, depending on available computational resources. Manual input time is well below that in any case.

While performance overall was not perfect, it seems highly adequate to answer clinically relevant questions: Did the ventricular volume increase or decrease? Are lateral ventricles and temporal horns wider or not? These questions are generally compared manually with linear measurements. Especially in ICU-settings with multiple scans in short periods, subtle changes can be difficult to objectify, especially with inconsistent positioning of patients. With detailed automated volumetry, we hope to provide additional insight into changes over time.

The additional graphical user interface should facilitate application in clinical settings. Also, we hope to lay the foundation for radiomic or topological analysis of shape features, such as the sphericity of frontal horns or the width of temporal horns (Han et al., 2022; Lo et al., 2021). While the resolution is currently kept at 256 × 256 × 256 for computational efficiency, it can easily be adapted to native voxel size to increase accuracy. Generally, the segmentation offers the potential for analyzing different indexes that have also been proposed to answer clinical questions differently (Ryska et al., 2020; Yamada et al., 2015).

### 4.1 Limitations

Distortive factors, such as patients who underwent craniectomy, were not included in our dataset. Hence, predicting from scans including these properties might not be compatible with our software. Also, the external validation cohort only contained patients with normal pressure hydrocephalus. Therefore, the pipeline is not yet ready to be applied to the entire spectrum of pathologies that require assessment of ventricular width. Yet, the current models can be used to speed up the creation of a new dataset. From a suggestion of the pipeline's output, manual correction can ensure a high-quality ground truth with reduced effort.

Limited by the graphical processing unit (GPU) on most clinical workstations, the calculation of the prediction can take several minutes. However, no action is required after the input and the task can be executed in the background. This makes it a time-efficient process.

Interrater agreement of neurosurgeons and radiologists for ventricular volume is not perfect, with an ICC of 0.846 (Bao et al., 2016). On the one hand, this creates a limiting factor of how well machine learning algorithms can approximate the absolute truth. They can only be as good as their input data which usually contains errors or disagreements rooting from manual segmentations (De Sutter et al., 2025; Karimi et al., 2020). On the other hand, with an 95% CI of ICC of up to 0.94–1.0 our models seem to have a better correlation to manual segmentation than the two human groups mentioned before. Additionally, they predict in a more objective manner, every time identical and are not subject to human variance.

Finally, further research and clinical evaluation is needed in order to assess the clinical benefits of computerized volumetric segmentation of ventricles.

## 5 Conclusion

Our pipeline is able to quantify the ventricular volume with anatomical subregions. This addresses a frequent issue in clinical practice since ventricular volume plays a role in a plethora of neurosurgical pathologies. With automated volumetry of anatomical subregions, we are able to offer the benefits of three-dimensional measurements in an objective manner as well as detailed analysis of regional variation in ventricular volumes, e.g. temporal horn changes only.

## CRediT authorship contribution statement

**Raffaele Da Mutten:** Writing – original draft, Visualization, Validation, Project administration, Methodology, Investigation, Formal analysis, Data curation, Conceptualization. **Olivier Zanier:** Writing – review & editing, Formal analysis, Data curation, Conceptualization. **Alessandro Carretta:** Writing – review & editing, Data curation. **Giorgio Palandri:** Writing – review & editing. **Massimo Bottini:** Writing – review & editing, Software, Project administration, Formal analysis, Data curation. **Daniel de Wilde:** Writing – review & editing, Software, Data curation. **Ulf C. Schneider:** Writing – review & editing, Supervision. **Luca Regli:** Writing – review & editing, Resources. **Carlo Serra:** Writing – review & editing, Project administration, Formal analysis, Conceptualization. **Victor E. Staartjes:** Writing – review & editing, Supervision, Project administration, Investigation, Formal analysis, Data curation, Conceptualization.

## Declaration of generative AI in writing

During the preparation of this work the authors used ChatGPT and Grammarly in order to improve language. After using this tool, the authors reviewed and edited the content as needed and take full responsibility for the content of the published article.

## Availability of data and material

The data in support of our findings can be obtained upon reasonable request from the corresponding author. Visit https://github.com/raffaele7/Ventricle/ in order to download the graphical user interface.

## Funding

Dr. Staartjes is supported by the Prof. Dr. Max Cloetta foundation. Otherwise, the authors declare that no funds, grants, or other support were received during the preparation of this manuscript.

## Declaration of competing interest

The authors declare that they have no known competing financial interests or personal relationships that could have appeared to influence the work reported in this paper.
